# Promoting equality, diversity and inclusion in research and funding: reflections from a digital manufacturing research network

**DOI:** 10.1186/s41073-024-00144-w

**Published:** 2024-05-16

**Authors:** Oliver J. Fisher, Debra Fearnshaw, Nicholas J. Watson, Peter Green, Fiona Charnley, Duncan McFarlane, Sarah Sharples

**Affiliations:** 1 Food, Water, Waste Research Group, Faculty of Engineering, University of Nottingham, University Park, Nottingham, UK; 2 Human Factors Research Group, Faculty of Engineering, University of Nottingham, University Park, Nottingham, UK; 3 School of Food Science and Nutrition, University of Leeds, Leeds, UK; 4 School of Engineering, University of Liverpool, Liverpool, UK; 5 Centre for Circular Economy, University of Exeter, Exeter, UK; 6 Institute for Manufacturing, University of Cambridge, Cambridge, UK

**Keywords:** Equality, Diversity, Inclusion, Research integrity, Network policy, Funding reviewing, EDI interventions

## Abstract

**Background:**

Equal, diverse, and inclusive teams lead to higher productivity, creativity, and greater problem-solving ability resulting in more impactful research. However, there is a gap between equality, diversity, and inclusion (EDI) research and practices to create an inclusive research culture. Research networks are vital to the research ecosystem, creating valuable opportunities for researchers to develop their partnerships with both academics and industrialists, progress their careers, and enable new areas of scientific discovery. A feature of a network is the provision of funding to support feasibility studies – an opportunity to develop new concepts or ideas, as well as to ‘fail fast’ in a supportive environment. The work of networks can address inequalities through equitable allocation of funding and proactive consideration of inclusion in all of their activities.

**Methods:**

This study proposes a strategy to embed EDI within research network activities and funding review processes. This paper evaluates 21 planned mitigations introduced to address known inequalities within research events and how funding is awarded. EDI data were collected from researchers engaging in a digital manufacturing network activities and funding calls to measure the impact of the proposed method.

**Results:**

Quantitative analysis indicates that the network’s approach was successful in creating a more ethnically diverse network, engaging with early career researchers, and supporting researchers with care responsibilities. However, more work is required to create a gender balance across the network activities and ensure the representation of academics who declare a disability. Preliminary findings suggest the network’s anonymous funding review process has helped address inequalities in funding award rates for women and those with care responsibilities, more data are required to validate these observations and understand the impact of different interventions individually and in combination.

**Conclusions:**

In summary, this study offers compelling evidence regarding the efficacy of a research network's approach in advancing EDI within research and funding. The network hopes that these findings will inform broader efforts to promote EDI in research and funding and that researchers, funders, and other stakeholders will be encouraged to adopt evidence-based strategies for advancing this important goal.

**Supplementary Information:**

The online version contains supplementary material available at 10.1186/s41073-024-00144-w.

## Introduction

Achieving equality, diversity, and inclusion (EDI) is an underpinning contributor to human rights, civilisation and society-wide responsibility [1]. Furthermore, promoting and embedding EDI within research environments is essential to make the advancements required to meet today’s research challenges [2]. This is evidenced by equal, diverse and inclusive teams leading to higher productivity, creativity and greater problem-solving ability [3], which increases the scientific impact of research outputs and researchers [4]. However, there remains a gap between EDI research and the everyday implementation of inclusive practices to achieve change [5]. This paper presents and reflects on the EDI measures trialled by the UK Engineering and Physical Sciences Research Council (EPSRC) funded digital manufacturing research network, Connected Everything (grant number: EP/S036113/1) [6]. The EPSRC is a UK research council that funds engineering and physical sciences research. By sharing these reflections, this work aims to contribute to the wider effort of creating an inclusive research culture. The perceptions of equality, diversity, and inclusion may vary among individuals. For the scope of this study, the following definitions are adopted:

- Equality: Equality is about ensuring that every individual has an equal opportunity to make the most of their lives and talents. No one should have poorer life chances because of the way they were born, where they come from, what they believe, or whether they have a disability.
- Diversity: Diversity concerns understanding that each individual is unique, recognising our differences, and exploring these differences in a safe, positive, and nurturing way to value each other as individuals.
- Inclusion: Inclusion is an effort and practice in which groups or individuals with different backgrounds are culturally and socially accepted, welcomed and treated equally. This concerns treating each person as an individual, making them feel valued, and supported and being respectful of who they are.

Research networks have varied goals, but a common purpose is to create new interdisciplinary research communities, by fostering interactions between researchers and appropriate scientific, technological and industrial groups. These networks aim to offer valuable career progression opportunities for researchers, through access to research funding, forming academic and industrial collaborations at network events, personal and professional development, and research dissemination. However, feedback from a 2021 survey of 19 UK research networks, suggests that these research networks are not always diverse, and whilst on the face of it they seem inclusive, they are perceived as less inclusive by minority groups (including non-males, those with disabilities, and ethnic minority respondents) [7]. The exclusivity of these networks further exacerbates the inequality within the academic community as it prevents certain groups from being able to engage with all aspects of network activities.

Research investigating the causes of inequality and exclusivity has identified several suggestions to make research culture more inclusive, including improving diverse representation within event programmes and panels [8, 9]; ensuring events are accessible to all [10]; providing personalised resources and training to build capacity and increase engagement [11]; educating institutions and funders to understand and address the barriers to research [12]; and increasing diversity in peer review and funding panels [13]. Universities, research institutions and research funding bodies are increasingly taking responsibility to ensure the health of the research and innovation system and to foster inclusion. For example, the EPSRC has set out their own ‘Expectation for EDI’ to promote the formation of a diverse and inclusive research culture [14]. To drive change, there is an emphasis on the importance of measuring diversity and links to measured outcomes to benchmark future studies on how interventions affect diversity [5]. Further, collecting and sharing EDI data can also drive aspirations, provide a target for actions, and allow institutions to consider common issues. However, there is a lack of available data regarding the impact of EDI practices on diversity that presents an obstacle, impeding the realisation of these benefits and hampering progress in addressing common issues and fostering diversity and inclusion [5].

Funding acquisition is important to an academic’s career progression, yet funding may often be awarded in ways that feel unequal and/or non-transparent. The importance of funding in academic career progression means that, if credit for obtaining funding is not recognised appropriately, careers can be damaged, and, as a result of the lack of recognition for those who have been involved in successful research, funding bodies may not have a complete picture of the research community, and are unable to deliver the best value for money [15]. Awarding funding is often a key research network activity and an area where networks can have a positive impact on the wider research community. It is therefore important that practices are established to embed EDI consideration within the funding process and to ensure that network funding is awarded without bias. Recommendations from the literature to make the funding award process fairer include: ensuring a diverse funding panel; funders instituting reviewer anti-bias training; anonymous review; and/or automatic adjustments to correct for known biases [16]. In the UK, the government organisation UK Research and Innovation (UKRI), tasked with overseeing research and innovation funding, has pledged to publish data to enhance transparency. This initiative aims to furnish an evidence base for designing interventions and evaluating their efficacy. While the data show some positive signs (e.g., the award rates for male and female PI applicants were equal at 29% in 2020–21), Ottoline Leyser (UKRI Chief Executive) highlights the ‘persistent pernicious disparities for under-represented groups in applying for and winning research funding’ [17]. This suggests that a more radical approach to rethinking the traditional funding review process may be required.

This paper describes the approach taken by the ‘Connected Everything’ EPSRC-funded Network to embed EDI in all aspects of its research funding process, and evaluates the impact of this ambition, leading to recommendations for embedding EDI in research funding allocation.

## Methods

### Connected everything’s equality diversity and inclusion strategy

Connected Everything aims to create a multidisciplinary community of researchers and industrialists to address key challenges associated with the future of digital manufacturing. The network is managed by an investigator team who are responsible for the strategic planning and, working with the network manager, to oversee the delivery of key activities. The network was first funded between 2016–2019 (grant number: EP/P001246/1) and was awarded a second grant (grant number: EP/S036113/1). The network activities are based around three goals: building partnerships, developing leadership and accelerating impact.

The Connected Everything network represents a broad range of disciplines, including manufacturing, computer science, cybersecurity, engineering, human factors, business, sociology, innovation and design. Some of the subject areas, such as Computer Science and Engineering, tend to be male-dominated (e.g., in 2021/22, a total of 185,42 higher education student enrolments in engineering & technology subjects was broken down as 20.5% Female and 79.5% Male [18]). The networks also face challenges in terms of accessibility for people with care responsibilities and disabilities. In 2019, Connected Everything committed to embedding EDI in all its network activities and published a guiding principle and goals for improving EDI (see Additional file 1). When designing the processes to deliver the second iteration of Connected Everything, the team identified several sources of potential bias/exclusion which have the potential to impact engagement with the network. Based on these identified factors, a series of mitigation interventions were implemented and are outlined in Table 1.

**Table 1:** Connected Everything (CE) planned mitigations (M) and intended outcomes to resolve equality, diversity and inclusion (EDI) factors effecting higher education research ecosystem

Factor	Reason	Evidence	Planned mitigation (M)	Intended outcome
Dominance of small number of research intensive Universities receiving funding from network	‘Rich get richer’ – social capital within Universities already experienced with EPSRC funding provides tacit support for applicants, prompts to engage etc	54.5% of CEI (2016–2019) feasibility study and 74.1% of EPSRC funding awarded between 2015–2021 to Russell Group universities [19]	**M1** Wide publicity for network opportunities **M2** Q&A session before each funding call **M3** Anonymisation of reviewing process **M4** Inclusive design of panel pitch sessions	Increased representation of less research intensive universities compared with other EPSRC schemes
Dominance of successful applications from men	Smaller pool of women and non-binary in academic positions Dominance of pastoral/teaching roles for women meaning less time available for research proposals/eternal engagement [20]	In 2016–2017, EPSRC awarded fewer than 7% of all research grants to teams led by women and, on average, grants awarded to women were less than 40% of the sums received by their male colleagues [21]	**M3** Anonymisation of reviewing process **M5** Visible diversity (including gender) in panel pitch members **M6** Publish CE Principal and Goals to demonstrate commitment to equality and fairness **M7** Collected EDI from whole research team applying	Higher representation of women awarded CE funding compared with other EPSRC schemes
Over-representation of people identifying as male in engineering and technology academic community	Women continue to be underrepresented in engineering leading to a lack of role models encouraging women to study engineering, which in turns increases the gender gap within the engineering workforce	In 2020/2021, 21.4% of UK engineering and technology related academic positions occupied by women [22] In 2020/2021, only 18% of those studying undergraduate degrees in engineering and technology are female [23]	**M6** Publish CE Principal and Goals to give confidence the network is inclusive **M8** Visible gender diversity in project leadership teams at all events **M9** Consideration of gendered language **M10** Collected EDI from individual engaging with CE events	Higher representation of women at network events compared to UK engineering and technology academic community
Dominance of successful applications from white academics	Lead applicants who identify in funding applications as an ‘ethnic minority’ have been less successful in their grant applications than those who identify as ‘white’	In 2018/19, the average success rate is 25 ± 1%, for ‘ethnic minority’ researchers as compared to 33 ± 2% for ‘white’ researchers, when submitting proposals for EPSRC funding [16]	**M3** Anonymisation of reviewing process **M4** Inclusive design of panel pitch sessions **M5** Visible diversity (including ethnicity) in panel pitch members **M7** Collected EDI from whole research team applying **M6** Publish CE Principal and Goals to give confidence give confidence to underrepresented ethnicities applying **M11** Ensure diversity in proposal reviewers	Higher representation of black/minority ethnic backgrounds awarded CE funding compared with other EPSRC schemes
Under-representation of those from black or minority ethnic backgrounds	Higher education within the UK is predominantly occupied by white academics, with academics from ethnic minority backgrounds facing structural, organisational and cultural barriers to career progression [24]	Between 2016/2017–2018/2019 of the total 19,868 PhD funded studentships awarded by UKRI research councils collectively, only 30 of those were from Black Caribbean backgrounds [25]	**M6** Publish CE Principal and Goals to give confidence the network is inclusive **M12** Visible ethnic diversity in presenters at all events **M13** Code of conduct at all events to show discriminator behaviour will not be tolerated	Higher representation of black/minority ethnic backgrounds at network events compared to UK engineering and technology academic community
Under-representation of disabilities, chronic conditions, invisible illnesses and neurodiversity in funded activities and events	Academics with disabilities continue to be under-represented in higher education People with disabilities faces additional barriers to attending events that has led to social exclusion, physical injuries and a desire never to attend future events [10]	The proportion of staff in universities declaring health conditions or impairments is 6% [26], while 21% of working-age adults [27] and nearly 19% of undergraduates [28] have a known disability	**M6** Publish CE Principal and Goals to give confidence the network is inclusive **M14** Inclusive design of network events with particular attention the needs of those with disabilities **M15** Ask attendees for any requirements that could require adjustments to make event accessible	Higher representation of those with declared disability or illness at network events compared to UK engineering and technology academic community Higher representation those with declared disability or illness awarded CE funding compared with other EPSRC schemes
Under-representation of those with care responsibilities in funded activities and events	Carers may face added physical, emotional and financial burdens. Managing the demands of paid and care work brings particular challenges for those in academic jobs, often characterised by heavy workloads and expectations of full availability	Staff reporting other caregiving responsibilities (besides childcare) were more likely to show probable depression (relative risk: 1.46) and anxiety (relative risk: 1.48) [29]	**M6** Publish CE Principal and Goals to give confidence the network is inclusive **M15** Ask attendees for any requirements that could require adjustments to make event accessible **M16** Inclusive design of network events with particular attention the needs of those with care responsibility	Collect and publish intersectional data about academics who are carers, as research on students and academics with care responsibilities remains scarce [30] Equal representation those with care responsibilities awarded CE funding
Reduced opportunities for ECRs	ECRs have been disproportionally affected by the COVID-19 pandemic and they bear the brunt of the burden of the pandemic-incurred hardships	ECRs have reported feeling insufficient support from their colleagues, a loss of networks and collaborations, and fears about their job security [29, 31]	**M17** Host ECR support forum **M18** Create ECR mentoring scheme	Higher representation of ECRs at network events compared to UK engineering and technology academic community
High competition for funding has greater impact on ECRs	Funding success rates for all career stages are low, but the burden falls most heavily on ECRs, who have a shorter track record [32]. Additionally, there is increased competition for limited funding, which can make it difficult for ECRs secure the resources necessary to pursue their research projects	In the UK, the award rate for Wellcome Trust fellowships and grants has declined from 19 to 12% in the between 2013–2018. The cause is most likely due to there being 37% more applicants as well as a 3% decrease in the number of awards [33]	**M2** Q&A session before each funding call **M3** Anonymisation of reviewing process **M19** Require ECRs within feasibility study project team **M20** Provide COVID recovery funding for ECRs **M21** ECR training webinar series	Higher representation of ECRs awarded CE funding compared with other EPSRC schemes

#### Connected everything anonymous review process

A key Connected Everything activity is the funding of feasibility studies to enable cross-disciplinary, foresight, speculative and risky early-stage research, with a focus on low technology-readiness levels. Awards are made via a short, written application followed by a pitch to a multidisciplinary diverse panel including representatives from industry. Six- to twelve-month-long projects are funded to a maximum value of £60,000.

**Fig. 1:**
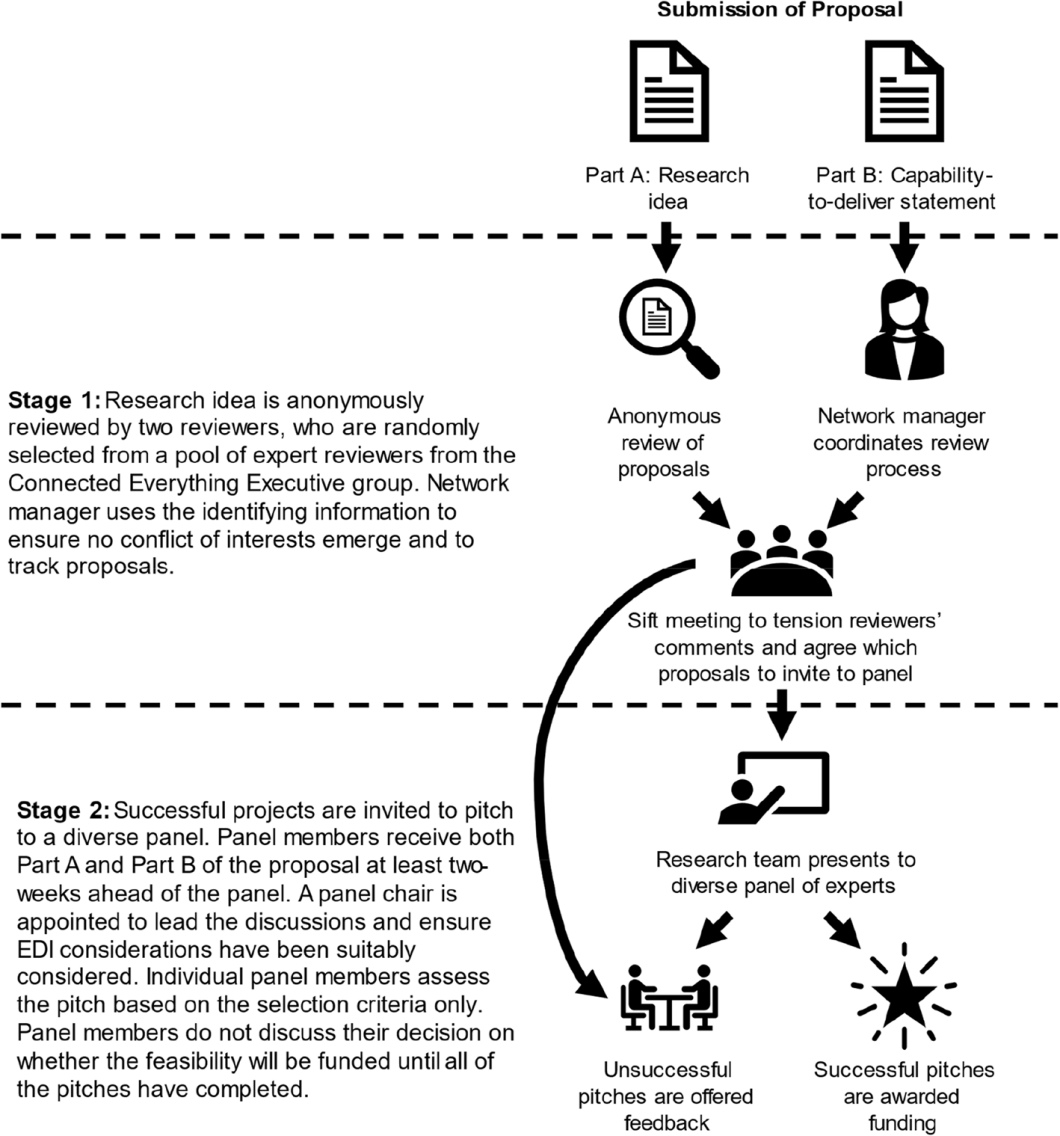
Connected Everything’s anonymous review process [EDI: Equality, diversity, and inclusion]

The current peer-review process used by funders may reveal the applicants’ identities to the reviewer. This can introduce dilemmas to the reviewer regarding (a) deciding whether to rely exclusively on information present within the application or search for additional information about the applicants and (b) whether or not to account for institutional prestige [34]. Knowing an applicant’s identity can bias the assessment of the proposal, but by focusing the assessment on the science rather than the researcher, equality is more frequently achieved between award rates (i.e., the proportion of successful applications) [15]. To progress Connected Everything’s commitment to EDI, the project team created a 2-stage review process, where the applicants’ identity was kept anonymous during the peer review stage. This anonymous process, which is outlined in Fig. 1, was created for the feasibility study funding calls in 2019 and used for subsequent funding calls.

To facilitate the anonymous review process, the proposal was submitted in two parts: part A the research idea and part B the capability-to-deliver statement. All proposals were first anonymously reviewed by a random selection of two members from the Connected Everything executive group, which is a diverse group of digital manufacturing experts and peers from academia, industry and research institutions that provide guidance and leadership on Connected Everything activities. The reviewers rated the proposals against the selection criteria (see Additional file 1, Table 1) and provided overall comments alongside a recommendation on whether or not the applicant should be invited to the panel pitch. This information was summarised and shared with a moderation sift panel, made up of a minimum of two Connected Everything investigators and a minimum of one member of the executive group, that tensioned the reviewers’ comments (i.e. comments and evaluations provided by the peer reviewers are carefully considered and weighed against each other) and ultimately decided which proposals to invite to the panel. This tension process included using the identifying information to ensure the applicants did have the capability to deliver the project. If this remained unclear, the applicants were asked to confirm expertise in an area the moderation sift panel thought was key or asked to bring in additional expertise to the project team during the panel pitch.

During stage two the applicants were invited to pitch their research idea to a panel of experts who were selected to reflect the diversity of the community. The proposals, including applicants’ identities, were shared with the panel at least two weeks ahead of the panel. Individual panel members completed a summary sheet at the end of the pitch session to record how well the proposal met the selection criteria (see Additional file 1, Table 1). Panel members did not discuss their funding decision until all the pitches had been completed. A panel chair oversaw the process but did not declare their opinion on a specific feasibility study unless the panel could not agree on an outcome. The panel and panel chair were reminded to consider ways to manage their unconscious bias during the selection process.

Due to the positive response received regarding the anonymous review process, Connected Everything extended its use when reviewing other funded activities. As these awards were for smaller grant values (~ £5,000), it was decided that no panel pitch was required, and the researcher’s identity was kept anonymous for the entire process.

### Data collection and analysis methods

#### Data collection

Equality, diversity and inclusion data were voluntarily collected from applicants for Connected Everything research funding and from participants who won scholarships to attend Connected Everything funded activities. Responses to the EDI data requests were collected from nine Connected Everything coordinated activities between 2019 and 2022. Data requests were sent after the applicant had applied for Connected Everything funding or had attended a Connected Everything funded activity. All data requests were completed voluntarily, with reassurance given that completion of the data requested in no way affected their application. In total 260 responses were received, of which the three feasibility study calls comprised 56.2% of the total responses received. Overall, there was a 73.8% response rate.

To understand the diversity of participants engaging with Connected Everything activities and funding, the data requests asked for details of specific diversity characteristics: gender, transgender, disability, ethnicity, age, and care responsibilities. Although sex and gender are terms that are often used interchangeably, they are two different concepts. To clarify, the definitions used by the UK government describe sex as a set of biological attributes that is generally limited to male or female, and typically attributed to individuals at birth. In contrast, gender identity is a social construction related to behaviours and attributes, and is self-determined based on a person’s internal perception, identification and experience. Transgender is a term used to describe people whose gender identity is not the same as the sex they were registered at birth. Respondents were first asked to identify their gender and then whether their gender was different from their birth sex.

For this study, respondents were asked to (voluntarily) self-declare whether they consider themselves to be disabled or not. Ethnicity within the data requests was based on the 2011 census classification system. When reporting ethnicity data, this study followed the AdvanceHE example to aggregate the census categories into six groups to enable benchmarking against the available academic ethnicity data. AdvanceHE is a UK charity that works to improve the higher education system for staff, students and society. However, it was acknowledged that there were limitations with this grouping, including the assumption that minority ethnic staff or students are a homogenous group [16]. Therefore, this study made sure to breakdown these groups during the discussion of the results. The six groups are:

1. Asian: Asian/Asian British: Indian, Pakistani, Bangladeshi, and any other Asian background;
2. Black: Black/African/Caribbean/Black British: African, Caribbean, and any other Black/African/Caribbean background;
3. Chinese;
4. Mixed;
5. Other ethnic backgrounds, including Arab.
6. White: all white ethnic groups.

#### Benchmarking data

Published data from the Higher Education Statistics Agency [26] (a UK organisation responsible for collecting, analysing, and disseminating data related to higher education institutions and students), UKRI funding data [19, 35] and 2011 census data [36] were used to benchmark the EDI data collected within this study. The responses to the data collected were compared to the engineering and technology cluster of academic disciplines, as this is most represented by Connected Everything’s main funded EPSRC. The Higher Education Statistics Agency defines the engineering and technology cluster as including the following subject areas: general engineering; chemical engineering; mineral, metallurgy & materials engineering; civil engineering; electrical, electronic & computer engineering; mechanical, aero & production engineering and; IT, systems sciences & computer software engineering [37].

When assessing the equality in funding award rates, previous studies have focused on analysing the success rates of only the principal investigators [15, 16, 38]; however, Connected Everything recognised that writing research proposals is a collaborative task, so requested diversity data from the whole research team. The average of the last six years of published principal investigator and co-investigator diversity data for UKRI and EPSRC funding awards (2015–2021) was used to benchmark the Connected Everything funding data [35]. The UKRI and EPSRC funding review process includes a peer review stage followed by panel pitch and assessment stage; however, the applicant's track record is assessed during the peer review stage, unlike the Connected Everything review process.

## Results

The data collected have been used to evaluate the success of the planned migrations to address EDI factors affecting the higher education research ecosystem, as outlined in Table 1 ("Connected Everything’s Equality Diversity and Inclusion Strategy" Section).

### Dominance of small number of research-intensive universities receiving funding from network

**Fig. 2:**
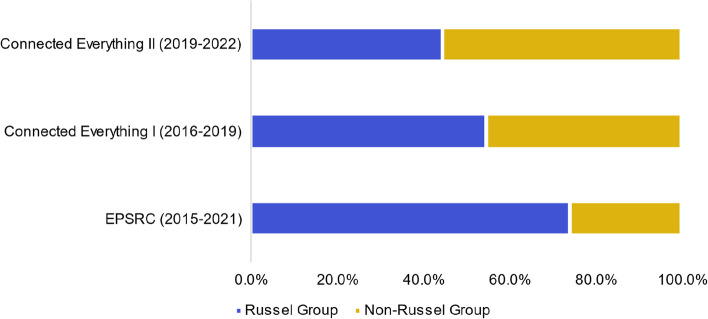
A comparison of funding awarded by EPSRC (total = £3.98 billion) across Russell Group universities and non-Russell Group universities, alongside the allocations for Connected Everything I (total = £660 k) and Connected Everything II (total = £540 k)

The dominance of a small number of research-intensive universities receiving funding from a network can have implications for the field of research, including: the unequal distribution of resources; a lack of diversity of research, limited collaboration opportunities; and impact on innovation and progress. Analysis of published EPSRC funding data between 2015 and 2021 [19], shows that the funding has been predominately (74.1%, 95% CI [71.%, 76.9%] out of £3.98 billion) awarded to Russell Group universities. The Russell Group is a self-selected association of 24 research-intensive universities (out of the 174 universities) in the UK, established in 1994. Evaluation of the universities that received Connected Everything feasibility study funding between 2016–2019, shows that Connected Everything awarded just over half (54.6%, 95% CI [25.1%, 84.0%] out of 11 awards) to Russell Group universities. Figure 2 shows that the Connected Everything funding awarded to Russell Group universities reduced to 44.4%, 95% CI [12.0%, 76.9%] of 9 awards between 2019–2022.

### Dominance of successful applications from men

**Fig. 3:**
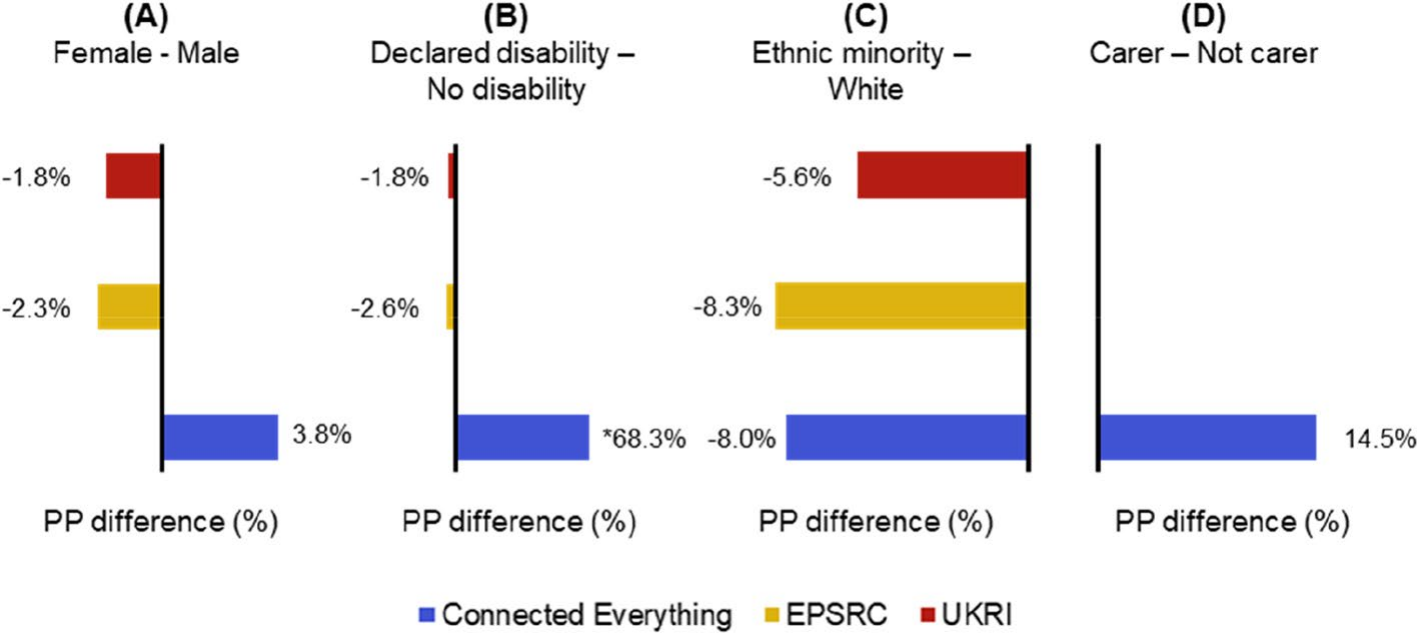
Percentage point (PP) differences in award rate by funding provider for gender, disability status, ethnicity and care responsibilities (data not collected by UKRI and EPSRC [35]). The total number of applicants for each funder are as follows: Connected Everything = 146, EPSRC = 37,960, and UKRI = 140,135. *The numbers of applicants were too small (< 5) to enable a meaningful discussion

The percentage point difference between the award rates of researchers who identified as female, those who declare a disability, or identified as ethnic minority applicants and carers and their respective counterparts have been plotted in Fig. 3. Bars to the right of the axis mean that the award rate of the female/declared-disability/ethnic-minority/carer applicants is greater than that of male/non- disability/white/not carer applicants.

Figure 3(A) shows that between 2015 and 2021 research team applicants who identified as male had a higher award rate than those who identified as female when applying for EPSRC and wider UKRI research council funding. Connected Everything funding applicants who identified as female achieved a higher award rate (19.4%, 95% CI [6.5%, 32.4%] out of 146) compared to male applicants (15.6%, 95% CI [8.8%, 22.4%] out of 146). These data suggest that biases have been reduced by the Connected Everything review process and other mitigation strategies (e.g., visible gender diversity in panel pitch members and publishing CE principal and goals to demonstrate commitment to equality and fairness). This finding aligns with an earlier study that found gender bias during the peer review process, resulting in female investigators receiving less favourable evaluations than their male counterparts [15].

### Over-representation of people identifying as male in engineering and technology academic community

**Fig. 4:**
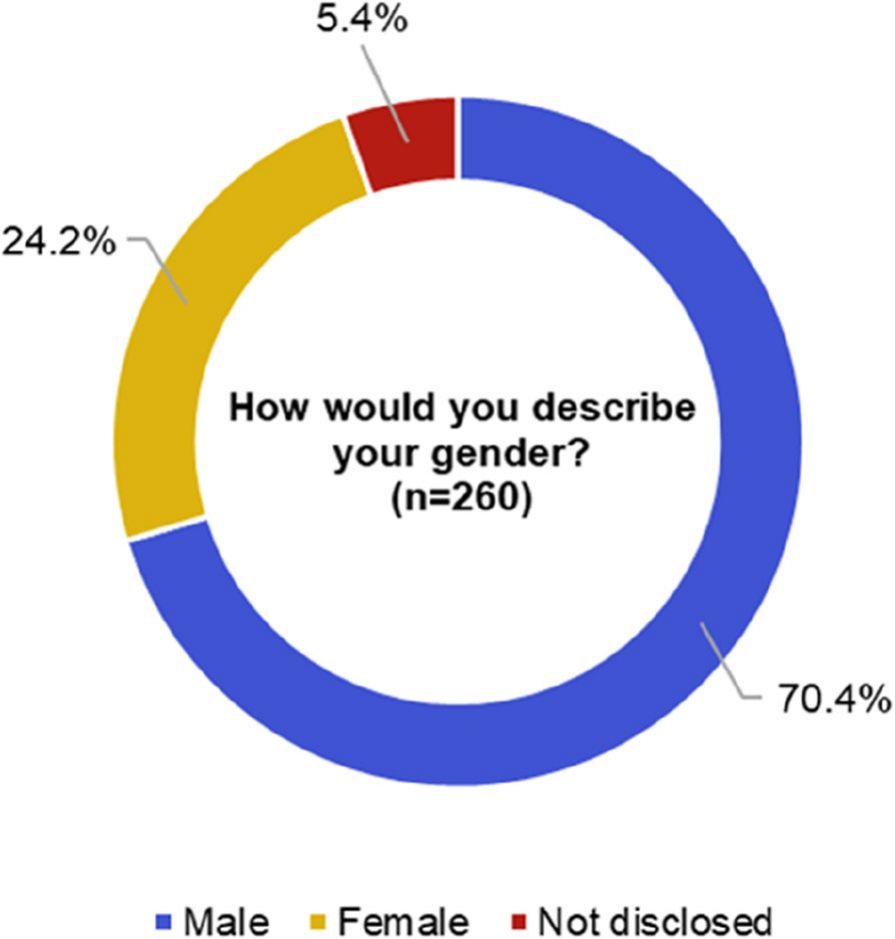
Gender question responses from a total of 260 respondents

Figure 4 shows the response to the gender question, with 24.2%, 95% CI [19.0%, 29.4%] of 260 responses identifying as female. This aligns with the average for the engineering and technology cluster (21.4%, 95% CI [20.9%, 21.9%] female of 27,740 academic staff), which includes subject areas representative of our main funder, EPSRC [22]. We also sought to understand the representation of transgender researchers within the network. However, following the rounding policy outlined by UK Government statistics policies and procedures [39], the number of responses that identified as a different sex to birth was too low (< 5) to enable a meaningful discussion.

### Dominance of successful applications from white academics

**Fig. 5:**
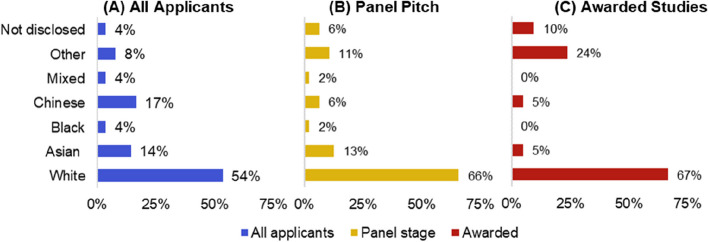
Ethnicity questions responses from different stages during the Connected Everything anonymous review process. The total number of applicants is 146, with 50 at the panel stage and 23 ultimately awarded

Figure 3(C) shows that researchers with a minority ethnicity consistently have a lower award rate than white researchers when applying for EPSRC and UKRI funding. Similarly, the results in Fig. 3(C) indicate that white researchers are more successful (8.0% percentage point, 95% CI [-8.6%, 24.6%]) when applying for Connected Everything funding. These results indicate that more measures should be implemented to support the ethnic minority researchers applying for Connected Everything funding, as well as sense checking there is no unconscious bias in any of the Connected Everything funding processes. The breakdown of the ethnicity diversity of applicants at different stages of the Connected Everything review process (i.e. all applications, applicants invited to panel pitch and awarded feasibility studies) has been plotted in Fig. 5 to help identify where more support is needed. Figure 5 shows an increase in the proportion of white researchers from 54%, 95% CI [45.4%, 61.8%] of all 146 applicants to 66%, 95% CI [52.8%, 79.1%] of the 50 researchers invited to the panel pitch. This suggests that stage 1 of the Connected Everything review process (anonymous review of written applications) may favour white applicants and/or introduce unconscious bias into the process.

### Under-representation of those from black or minority ethnic backgrounds

**Fig. 6:**
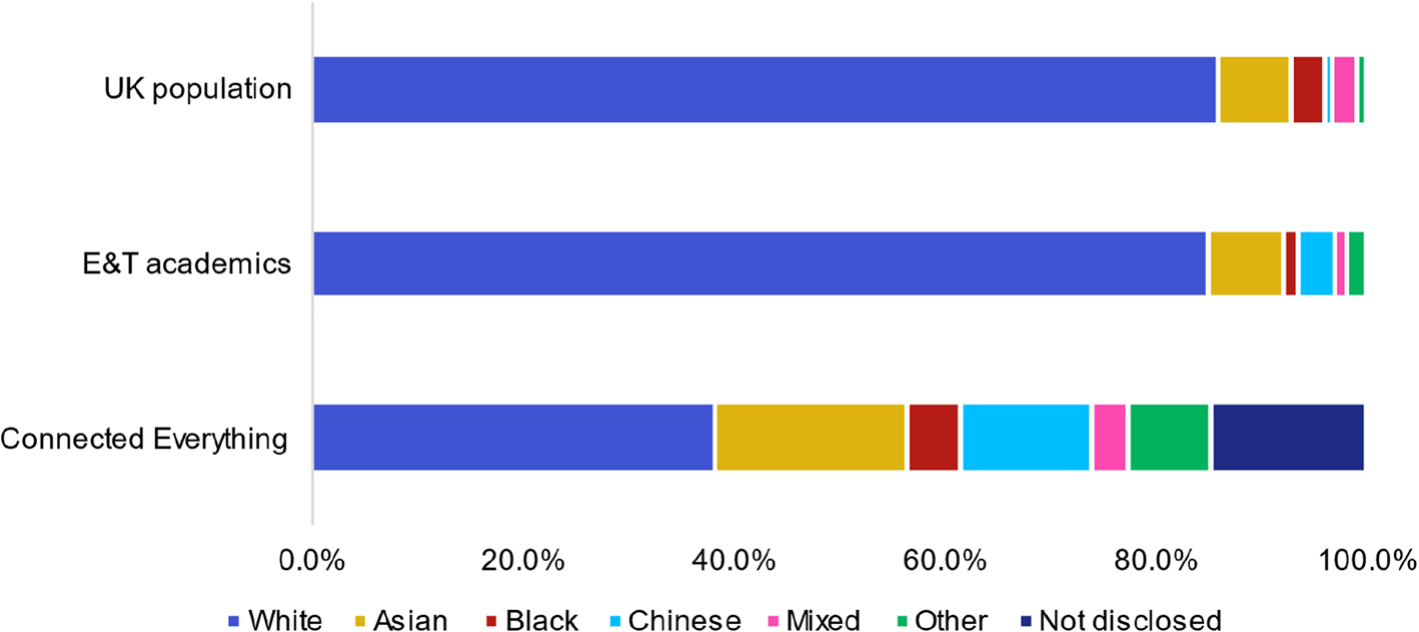
Ethnicity question responses from a total of 260 respondents compared to distribution of the 13,085 UK engineering and technology (E&T) academic staff [22] and 56 million people recorded in the UK 2011 census data [36]

Connected Everything appears to have a wide range of ethnic diversity, as shown in Fig. 6. The ethnicities Asian (18.3%, 95% CI [13.6%, 23.0%]), Black (5.1%, 95% CI [2.4%, 7.7%]), Chinese (12.5%, 95% CI [8.4%, 16.5%]), mixed (3.5%, 95% CI [1.3%, 5.7%]) and other (7.8%, 95% CI [4.5%, 11.1%]) have a higher representation among the 260 individuals engaging with network’s activities, in contrast to both the engineering and technology academic community and the wider UK population. When separating these groups into the original ethnic diversity answers, it becomes apparent that there is no engagement with ‘Black or Black British: Caribbean’, ‘Mixed: White and Black Caribbean’ or ‘Mixed: White and Asian’ researchers within Connected Everything activities. The lack of engagement with researchers from a Caribbean heritage is systemic of a lack of representation within the UK research landscape [25].

### Under-representation of disabilities, chronic conditions, invisible illnesses and neurodiversity in funded activities and events.

**Fig. 7:**
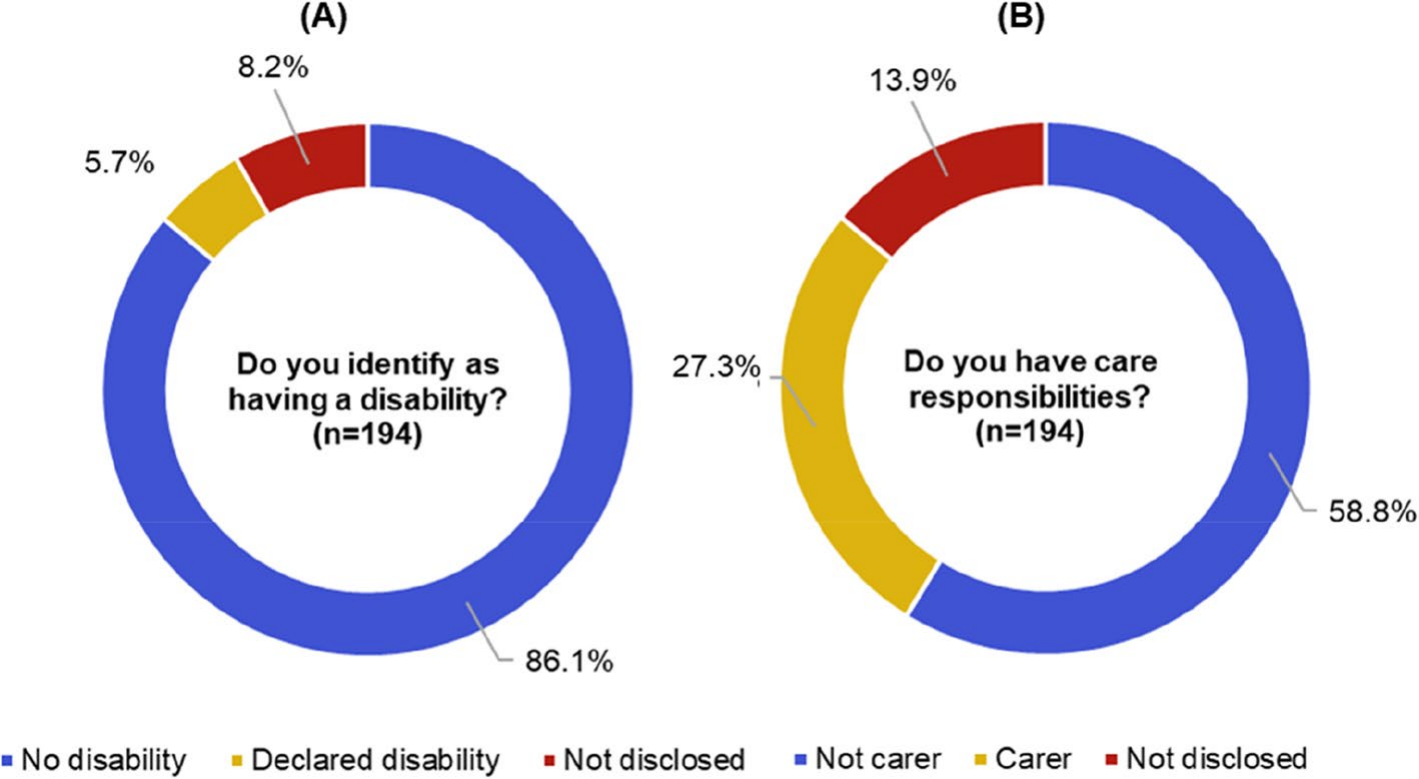
Responses to **A** Disability and **B** Care responsibilities questions colected from a total of 194 respondents

Figure 7(A) shows that 5.7%, 95% CI [2.4%, 8.9%] of 194 responses declared a disability. This is higher than the average of engineering and technology academics that identify as disabled (3.4%, 95% CI [3.2%, 3.7%] of 27,730 academics). Between Jan-March 2022, 9.0 million people of working age (16–64) within the UK were identified as disabled by the Office for National Statistics [40], which is 21% of the working age population [27]. Considering these statistics, there is a stark under-representation of disabilities, chronic conditions, invisible illnesses and neurodiversity amongst engineering and technology academic staff and those engaging in Connected Everything activities.

Between 2015 and 2020 academics that declared a disability have been less successful than academics without a disability in attracting UKRI and EPSRC funding, as shown in Fig. 3(B). While Fig. 3(B) shows that those who declare a disability have a higher Connected Everything funding award rate, the number of applicants who declared a disability was too small (< 5) to enable a meaningful discussion regarding this result.

### Under-representation of those with care responsibilities in funded activities and events

**Fig. 8:**
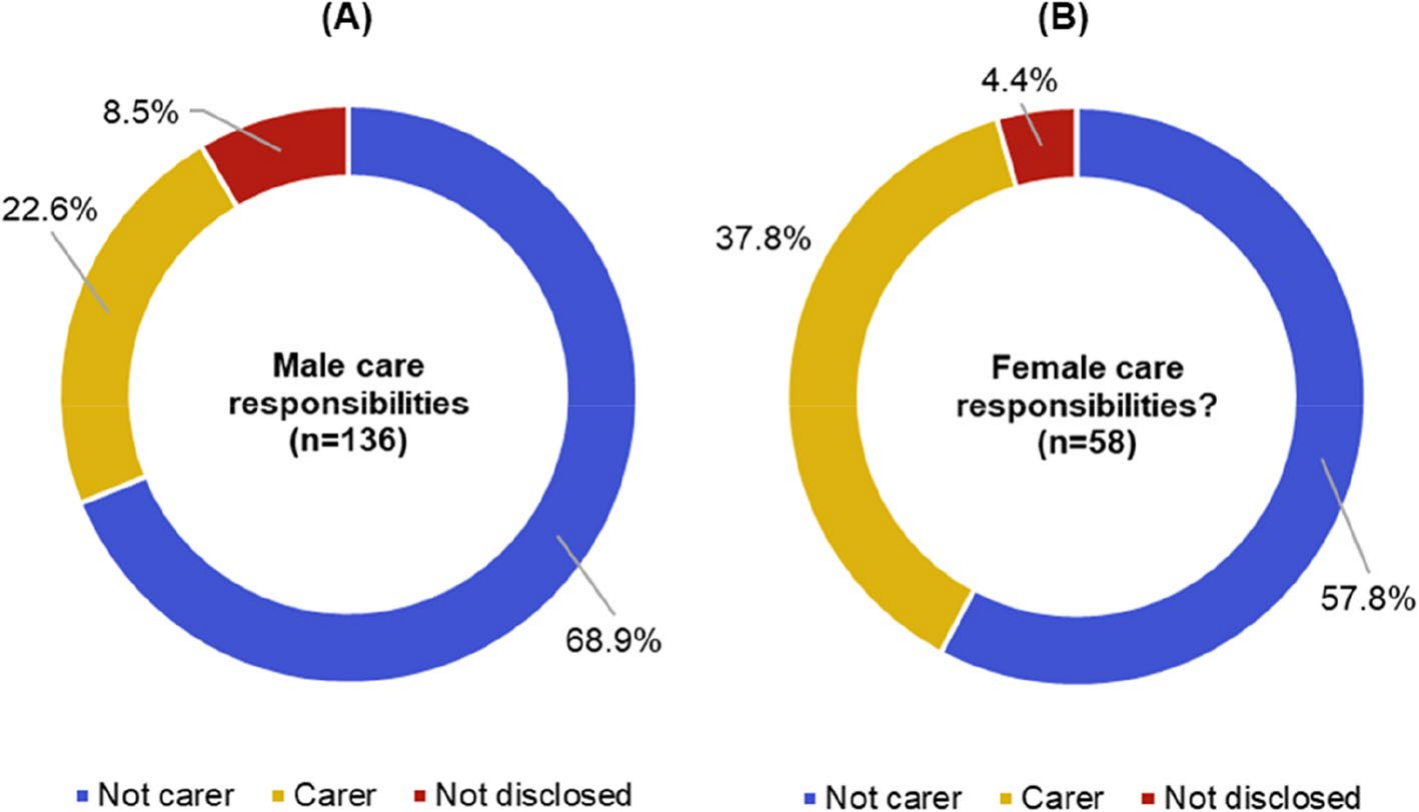
Responses to care responsibilities when grouped by **A** 136 males and **B** 58 females

In response to the care responsibilities question, Fig. 7(B) shows that 27.3%, 95% CI [21.1%, 33.6%] of 194 respondents identified as carers, which is higher than the 6% of adults estimated to be providing informal care across the UK in a UK Government survey of the 2020/2021 financial year [41]. However, the ‘informal care’ definition used by the 2021 survey includes unpaid care to a friend or family member needing support, perhaps due to illness, older age, disability, a mental health condition or addiction [41]. The Connected Everything survey included care responsibilities across the spectrum of care that includes partners, children, other relatives, pets, friends and kin. It is important to consider a wide spectrum of care responsibilities, as key academic events, such as conferences, have previously been demonstrably exclusionary sites for academics with care responsibilities [42]. Breakdown analysis of the responses to care responsibilities by gender in Fig. 8 reveals that 37.8%, 95% CI [25.3%, 50.3%] of 58 women respondents reported care responsibilities, compared to 22.6%, 95% CI [61.1%, 76.7%] of 136 men respondents. Our findings reinforce similar studies that conclude the burden of care falls disproportionately on female academics [43].

Figure 3(D) shows that researchers with careering responsibilities applying for Connected Everything funding have a higher award rate than those researchers applying without care responsibilities. These results suggest that the Connected Everything review process is supportive of researchers with care responsibilities, who have faced barriers in other areas of academia.

### Reduced opportunities for ECRs

Early-career researchers (ECRs) represent the transition stage between starting a PhD and senior academic positions. EPSRC defines an ECR as someone who is either within eight years of their PhD award, or equivalent professional training or within six years of their first academic appointment [44]. These periods exclude any career break, for example, due to family care; health reasons; and reasons related to COVID-19 such as home schooling or increased teaching load. The median age for starting a PhD in the UK is 24 to 25, while PhDs usually last between three and four years [45]. Therefore, these data would imply that the EPSRC median age of ECRs is between 27 and 37 years. It should be noted, however, that this definition is not ideal and excludes ECRs who may have started their research career later in life.

**Fig. 9:**
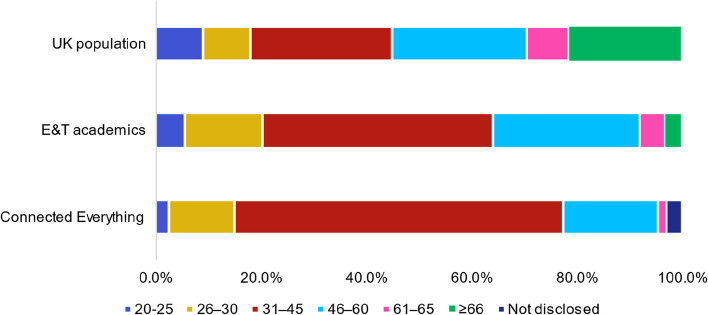
Age question responses from a total of 194 respondents compared to distribution of the 27,780 UK engineering and technology (E&T) academic staff [22] and 56 million people recorded in the UK 2011 census data [36]

Connected Everything aims to support ECRs via measures that include mentoring support, workshops, summer schools and podcasts. Figure 9 shows a greater representation of researchers engaging with Connected Everything activities that are aged between 30–44 (62.4%, 95% CI [55.6%, 69.2%] of 194 respondents) when compared to the wider engineering and technology academic community (43.7%, 95% CI [43.1%, 44.3%] of 27,780 academics) and UK population (26.9%, 95% CI [26.9%, 26.9%]).

### High competition for funding has a greater impact on ECRs

**Fig. 10:**
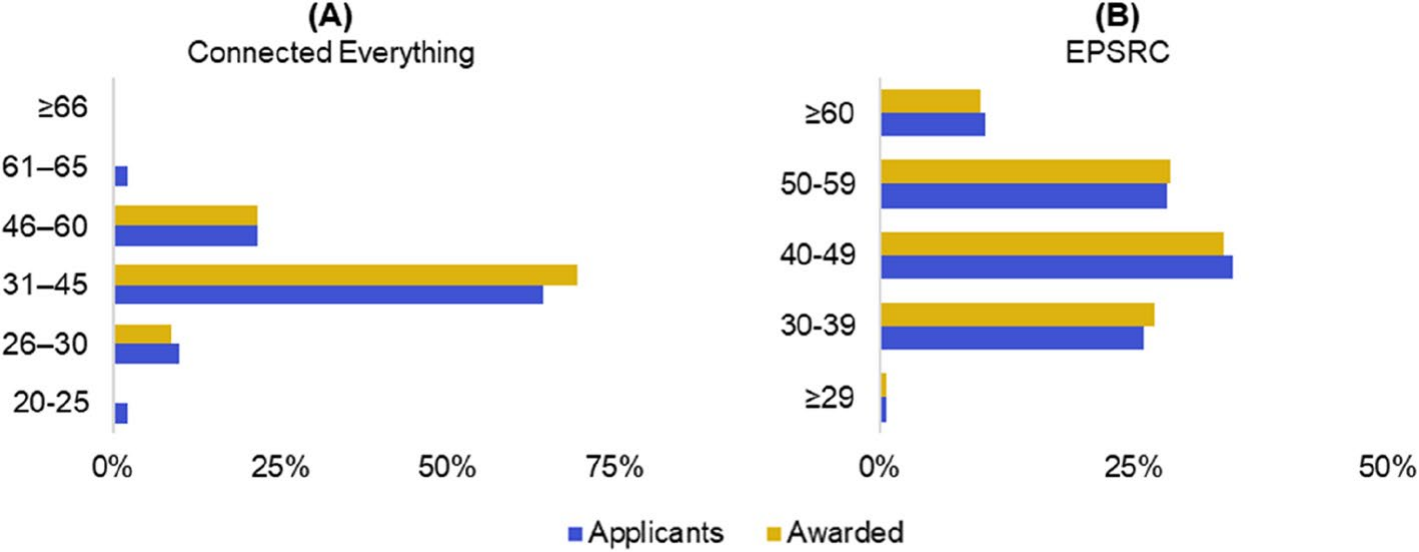
Age of researchers at applicant and awarded funding stages for **A** Connected Everything between 2019–2022 (total of 146 applicants and 23 awarded) and **B** EPSRC funding between 2015–2021 [35] (total of 35,780 applicants and 12,075 awarded)

Figure 10 shows that the largest age bracket applying for and winning Connected Everything funding is 31–45, whereas 72%, CI 95% [70.1%, 74.5%] of 12,075 researchers awarded EPSRC grants between 2015 and 2021 were 40 years or older. These results suggest that measures introduced by Connected Everything has been successful at providing funding opportunities for researchers who are likely to be early-mid career stage.

## Discussion

The results of this paper provide insights into the impact that Connected Everything’s planned mitigations have had on promoting equality, diversity, and inclusion (EDI) in research and funding. Collecting EDI data from individuals who engage with network activities and apply for research funding enabled an evaluation of whether these mitigations have been successful in achieving the intended outcomes outlined at the start of the study, as summarised in Table 2.

**Table 2:** Evaluating whether the Connected Everything (CE) mitigations achieved the desired outcome

Intended outcome	Result
Increased representation of less research-intensive universities compared with other EPSRC schemes	Connected Everything (CE) awarded 63.6% of funding to non-Russell Group universities compared to 25.9% of EPSRC funding awarded to non-Russell Group universities
Higher representation of women awarded CE funding compared with other EPSRC schemes	Female applicants award rates were 3.8 percentage points higher than their male counters parts when applying for CE funding whereas female applicants award was 2.3 percentage points lower for EPSRC funding between 2015–2021
Higher representation of women at network events compared to UK engineering and technology academic community	24.2% of responses identifying as female, which was higher than 21.4% of female staff that make up the engineering and technology academic staff within the UK
Higher representation of black/minority ethnic backgrounds awarded CE funding compared with other EPSRC schemes	In CE and EPSRC funding, white researchers award rates were 8 and 8.3 percentage points higher than ethnic minority researchers, respectively
Higher representation of black/minority ethnic backgrounds at network events compared to UK engineering and technology academic community	The ethnicities Asian (18.3%), Black (5.1%), Chinese (12.5%), mixed (3.5%) and other (7.8%) have a higher representation within CE network activities when compared to both the engineering and technology academic community (Asian: 7.2%; Black: 1.3%; Chinese: 3.4%; mixed 2.1%; other: 1.1%)
Higher representation of those with declared disability or illness at network events compared to UK engineering and technology academic community	A higher representation of those with declare disabilities in CE network activities (5.7%) compared to engineering and technology academic community
Higher representation those with declared disability or illness awarded CE funding compared with other EPSRC schemes	The number of applicants who declared a disability was too small (< 5) to enable a meaningful discussion regarding this result
Collect and publish intersectional data about academics who are carers, as research on students and academics with care responsibilities remains scarce	27.3% of people engaging with CE activities identified as carers. Breakdown analysis of the responses to care responsibilities by gender in reveals that 37.8% of women report care responsibilities, compared to 22.6% men
Equal representation those with care responsibilities awarded CE funding	Researchers with care responsibilities applying for CE funding have a higher award rate (26.8%) than those researchers applying without care responsibilities (12.3%)
Higher representation of ECRs at network events compared to UK engineering and technology academic community	Greater representation of researchers engaging with CE activities that are aged between 30–44 (62.4%) when compared to the wider engineering and technology academic community (43.7%) and UK population (26.9%)
Higher representation of ECRs awarded CE funding compared with other EPSRC schemes	70% of CE funding is awarded to applicants in the age range of 31–45, whereas 72% of EPSRC funding between 2015 and 2021 was won by researchers 40 years or older

The results in Table 2 indicate that Connected Everything’s approach to EDI has helped achieve the intended outcome to improve representation of women, ECRs, those with a declared disability and black/minority ethnic backgrounds engaging with network events when compared to the engineering and technology academic community. In addition, the network has helped raise awareness of the high presence of researchers with care responsibilities at network events, which can help to track progress towards making future events inclusive and accessible towards these carers. The data highlights two areas for improvement: (1) ensuring a gender balance; and (2) increasing representation of those with declared disabilities. Both these discrepancies are indicative of the wider imbalances and underrepresentation of these groups in the engineering and technology academic community [26], yet represent areas where networks can strive to make a difference. Possible strategies include: using targeted outreach; promoting greater representation of these groups in event speakers; and going further to create a welcoming and inclusive environment. One barrier that can disproportionately affect women researchers is the need to balance care responsibilities with attending network events [46]. This was reflected in the Connected Everything data that reported 37.8%, 95% CI [25.3%, 50.3%] of women engaging with network activities had care responsibilities, compared to 22.6%, 95% CI [61.1%, 76.7%] of men. Providing accommodations such as on-site childcare, flexible scheduling, or virtual attendance options can therefore help to promote inclusivity and allow more women researchers to attend.

Only 5.7%, 95% CI [2.4%, 8.9%] of responses engaging with Connected Everything declared a disability, which is higher than the engineering and technology academic community (3.4%, 95% CI [3.2%, 3.7%]) [26], but unrepresentative of the wider UK population. It has been suggested that academics can be uncomfortable when declaring disabilities because scholarly contributions and institutional citizenship are so prized that they feel they cannot be honest about their issues or health concerns and keep them secret [47]. In research networks, it is important to be mindful of this hidden group within higher education and ensure that measures are put in place to make the network’s activities inclusive to all. Future considerations for accommodations to improve research events inclusivity include: improving physical accessibility of events; providing assistive technology such as screen readers, audio descriptions, and captioning can help individuals with visual or hearing impairments to access and participate; providing sign language interpreters; offering flexible scheduling options; and the provision of quiet rooms, written materials in accessible formats, and support staff trained to work with individuals with cognitive disabilities.

Connected Everything introduced measures (e.g., anonymised reviewing process, Q&A sessions before funding calls, inclusive design of panel pitch) to help address inequalities in how funding is awarded. Table 2 shows success in reducing the dominance of researchers who identify as male and research-intensive universities in winning research funding and that researchers with care responsibilities were more successful at winning funding than those without care responsibilities. The data revealed that the proposed measures were unable to address the inequality in award rates between white and ethnic minority researchers, which is an area to look to improve. The inequality appears to occur during the anonymous review stage, with a greater proportion of white researchers being invited to panel. Recommendations to make the review process fairer include: ensuring greater diversity of reviewers; reviewer anti-bias training; and automatic adjustments to correct for known biases in writing style [16, 32].

When reflecting on the development of a strategy to embed EDI throughout the network, Connected Everything has learned several key lessons that may benefit other networks undergoing a similar activity. These include:

- EDI is never ‘done’: There is a constant need to review approaches to EDI to ensure they remain relevant to the network community. Connected Everything could review its principles to include the concept of justice in its approach to diversity and inclusion. The concept of justice concerning EDI refers to the removal of systematic barriers that stop fair and equitable distribution of resources and opportunities among all members of society, regardless of their individual characteristics or backgrounds. The principles and subsequent actions could be reviewed against the EDI expectations [14], paying particular attention to areas where barriers may still be present. For example, shifting from welcoming people into existing structures and culture to creating new structures and culture together, with specific emphasis on decision or advisory mechanisms within the network. This activity could lend itself to focusing more on tailored support to overcome barriers, thus achieving equity, if it is not within the control of the network to remove the barrier itself (justice).
- Widen diversity categories: By collecting data on a broad range of characteristics, we can identify and address disparities and biases that might otherwise be overlooked. A weakness of this dataset is that ignores the experience of those with intersectional identities, across race, ethnicity, gender, class, disability and/ or LGBTQI. The Wellcome Trust noted how little was known about the socio-economic background of scientists and researchers [48].
- Collect data on whole research teams: For the first two calls for feasibility study funding, Connected Everything only asked the Principal Investigator to voluntarily provide their data. We realised that this was a limited approach and, in the third call, asked for the data regarding the whole research team to be shared anonymously. Furthermore, we do not currently measure the diversity of our event speakers, panellists or reviewers. Collecting these data in the future will help to ensure the network is accountable and will ensure that all groups are represented during our activities and in the funding decision-making process.
- High response rate: Previous surveys measuring network diversity (e.g., [7]) have struggled to get responses when surveying their memberships; whereas, this study achieved a response rate of 73.8%. We attribute this high response rate to sending EDI data requests on the point of contact with the network (e.g., on submitting funding proposals or after attending network events), rather than trying to survey the entire network membership at anyone point in time.
- Improve administration: The administration associated with collecting EDI data requires a commitment to transparency, inclusivity, and continuous improvement. For example, during the first feasibility funding call, Connected Everything made it clear that the review process would be anonymous, but the application form was not in separate documents. This made anonymising the application forms extremely time-consuming. For the subsequent calls, separate documents were created – Part A for identifying information (Principal Investigator contact details, Project Team and Industry collaborators) and Part B for the research idea.
- Accepting that this can be uncomfortable: Trying to improve EDI can be uncomfortable because it often requires challenging our assumptions, biases, and existing systems and structures. However, it is essential if we want to make real progress towards equity and inclusivity. Creating processes to support embedding EDI takes time and Connected Everything has found it is rare to get it right the first time. Connected Everything is sharing its learning as widely as possible both to support others in their approaches and continue our learning as we reflect on how to continually improve, even when it is challenging.
- Enabling individual engagement with EDI: During this work, Connected Everything recognised that methods for engaging with such EDI issues in research design and delivery are lacking. Connected Everything, with support from the Future Food Beacon of Excellence at the University of Nottingham, set out to develop a card-based tool [49] to help researchers and stakeholders identify questions around how their work may promote equity and increase inclusion or have a negative impact towards one or more protected groups and how this can be overcome. The results of this have been shared at conference presentations [50] and will be published later.

While this study provides insights into how EDI can be improved in research network activities and funding processes, it is essential to acknowledge several limitations that may impact the interpretation of the findings.

- Sample size and generalisability: A total of 260 responses were received, which may not be representative of our overall network of 500 + members. Nevertheless, this data provides a sense of the current diversity engaging in Connected Everything activities and funding opportunities, which we can compare with other available data to steer action to further diversify the network.
- Handling of missing data: Out of the 260 responses, 66 data points were missing for questions regarding age, disability, and caring responsibilities. These questions were mistakenly omitted from a Connected Everything summer school survey, contributing to 62 missing data points. While we assumed the remainer of missing data to be at random during analysis, it's important to acknowledge it could be related to other factors, potentially introducing bias into our results.
- Emphasis on quantitative data: The study relies on using quantitative data to evaluate the impact of the EDI measures introduced by Connected Everything. However, relying solely on quantitative metrics may overlook nuanced aspects of EDI that cannot be easily quantified. For example, EDI encompasses multifaceted issues influenced by historical, cultural, and contextual factors. These nuances may not be fully captured by numbers alone. In addition, some EDI efforts may not yield immediate measurable outcomes but still contribute to a more inclusive environment.
- Diversity and inclusion are not synonymous: The study proposes 21 measures to contribute towards creating an equal, diverse and inclusive research culture and collects diversity data to measure the impact of these measures. However, while diversity is simpler to monitor, increasing diversity alone does not guarantee equality or inclusion. Even with diverse research groups, individuals from underrepresented groups may still face barriers, microaggressions, or exclusion.
- Balancing anonymity and rigour in grant reviews:The proposed anonymous review process proposed by Connected Everything removes personal and organisational details from the research ideas under reviewer evaluation. However, there exists a possibility that a reviewer could discern the identity of the grant applicant based on the research idea. Reviewers are expected to be subject matter experts in the field relevant to the grant proposal they are evaluating. Given the specialised nature of scientific research, it is conceivable that a well-known applicant could be identified through the specifics of the work, the methodologies employed, and even the writing style.
- Expanding gender identity options: A limitation of this study emerged from the restricted gender options (male, female, other, prefer not to say) provided to respondents when answering the gender identity question. This limitation reflects the context of data collection in 2018, a time when diversity monitoring guidance was still limited. As our understanding of gender identity evolves beyond binary definitions, future data collection efforts should embrace a more expansive and inclusive approach, recognising the diverse spectrum of gender identities.

## Conclusion

In conclusion, this study provides evidence of the effectiveness of a research network's approach to promoting equality, diversity, and inclusion (EDI) in research and funding. By collecting EDI data from individuals who engage with network activities and apply for research funding, this study has shown that the network's initiatives have had a positive impact on representation and fairness in the funding process. Specifically, the analysis reveals that the network is successful at engaging with ECRs, and those with care responsibilities and has a diverse range of ethnicities represented at Connected Everything events. Additionally, the network activities have a more equal gender balance and greater representation of researchers with disabilities when compared to the engineering and technology academic community, though there is still an underrepresentation of these groups compared to the national population.

Connected Everything introduced measures to help address inequalities in how funding is awarded. The measures introduced helped reduce the dominance of researchers who identified as male and research-intensive universities in winning research funding. Additionally, researchers with care responsibilities were more successful at winning funding than those without care responsibilities. However, inequality persisted with white researchers achieving higher award rates than those from ethnic minority backgrounds. Recommendations to make the review process fairer include: ensuring greater diversity of reviewers; reviewer anti-bias training; and automatic adjustments to correct for known biases in writing style.

Connected Everything’s approach to embedding EDI in network activities has already been shared widely with other EPSRC-funded networks and Hubs (e.g. the UKRI Circular Economy Hub and the UK Acoustics Network Plus). The network hopes that these findings will inform broader efforts to promote EDI in research and funding and that researchers, funders, and other stakeholders will be encouraged to adopt evidence-based strategies for advancing this important goal.

### Supplementary Information

**Supplementary Material 1.**

## Data Availability

The data collected was anonymously, however, it may be possible to identify an individual by combining specific records of the data request form data. Therefore, the study data has been presented in aggregate form to protect the confidential of individuals and the data utilised in this study cannot be made openly accessible due to ethical obligations to protect the privacy and confidentiality of the data providers.

## Abbreviations

ECR: Early career researcher
EDI: Equality, diversity and inclusion
EPSRC: Engineering physical sciences research council
UKRI: UK research and innovation
