# Supplementary material for: Promoting equality, diversity and inclusion in research and funding: reflections from a digital manufacturing research network
Source: Res Integr Peer Rev. 2024 May 16;9:5. doi: 10.1186/s41073-024-00144-w (PMC11097576; doi:10.1186/s41073-024-00144-w)
Supplement: Supplementary file 1 — Supplementary Material 1. [file 41073_2024_144_MOESM1_ESM.docx]

**Promoting Equality, Diversity and Inclusion in Research and Funding: Reflections from a Digital Manufacturing Research Network**

**Additional file 1**

Oliver J FISHER^1^, Debra FEARNSHAW^2*^, Nicholas J WATSON^3^, Peter GREEN^4^, Fiona CHARLEY^5^, Duncan MCFARLANE^6^, Sarah SHARPLES^2^

^1^ Food, Water, Waste Research Group, Faculty of Engineering, University of Nottingham, University Park, Nottingham, United Kingdom

^2^ Human Factors Research Group, Faculty of Engineering, University of Nottingham, University Park, Nottingham, United Kingdom

^3^ School of Food Science and Nutrition, University of Leeds, United Kingdom

^4^ School of Engineering, University of Liverpool, United Kingdom

^5^ Centre for Circular Economy, University of Exeter, United Kingdom

^6^ Institute for Manufacturing, University of Cambridge, United Kingdom

*Corresponding Author: [debra.fearnshaw@nottingham.ac.uk](mailto:debra.fearnshaw@nottingham.ac.uk)

**Table of Contents**

[**Connected Everything Equality, Diversity and Inclusion Principle (November 2020)** 2](#_Toc161397934)

[**Table 1** Connected Everything selection criteria for stage 1 and 2 of the anonymous review process. 5](#_Toc161397935)

# **Connected Everything Equality, Diversity and Inclusion Principle (November 2020)**

As Pro-Vice Chancellor for Equality, Diversity, Inclusion and People at the University of Nottingham, it will not surprise you that I am actively championing EDI across all the projects I lead. As Principal Investigator for Connected Everything, I am often helpfully challenged on why EDI is important. For me, it is simple. Without implementing EDI principles throughout our network, it will not be the best. We will not reach out to the breadth of talent available in the UK. Our research and activities will not be as rich and our research outcomes will be poorer. EDI is central to delivering high quality research outcomes. I am keen to ensure Connected Everything is fully representative of the digital manufacturing community it serves. These principles will help guide our way as we strive to achieve this.

***Sarah Sharples***

Pro-Vice-Chancellor for Equality, Diversity and Inclusion and People

Professor of Human Factors

Principal Investigator, Connected Everything

University of Nottingham

**The Principle**

*“Connected Everything knows that diverse teams deliver high quality research outcomes. Furthermore, Equality, Diversity and Inclusion approaches lead to happy and healthy research teams. CEII wants to encourage, support and respect ideas from everyone and ensure our inclusive activities are representative of our network’s community.”*

Connected Everything will use its Equality, Diversity and Inclusion (EDI) principle for all its activities to ensure that anyone who engages with Connected Everything is aware of our commitment.

This document will be implemented in January 2021 with sign off from the Project Team and given to the Executive Group for information, as well as being available on the Connected Everything website.

Progress against the goals, which underpin the principle, will be reported quarterly to the Project Team and the Executive Group Sarah Sharples and the investigator team will own this document. Debra Fearnshaw will develop an action plan to support implementation of the goals

**Our Goals**

Goals will be achieved within the Connected Everything network timescales until September 2022.

1. Connected Everything will collect monitoring data from participants / applicants for all the activities it offers.
   1. This data will be held anonymously
   2. Completion of data requests will be on a voluntary basis
   3. Connected Everything will move beyond the focus on the PI and from 2021 will collect data on all team members, including CIs, research staff, managers and technicians, embracing the notion of team science and will endeavour to collect this retrospectively from studies funded in 2020
   4. Connected Everything will use this data to inform its activities
2. From January 2021, where Connected Everything is funding an activity, we will require the applicant to produce a short statement on how EDI will be considered and implemented during the funded project.
3. Connected Everything will have an anonymous process for submitting feasibility proposals and individual applicants will remain anonymous up to the panel pitch stage of the assessment process.
4. Connected Everything will aim to achieve will aim to achieve a balance of participants across all its activities including:
   1. a gender balance for PIs leading funded feasibility studies
   2. at least 1 ECR holds a demonstrable research role in the feasibility study research team
   3. diverse ethnic representation for PIs leading funded feasibility studies.
   4. improve representation of people with a declared disability within the research team of funded feasibility studies
5. Connected Everything will develop an ECR Mentor Scheme to support their career development. The scheme will aim to support a minimum of 10 ECRs. Applications from under-represented groups will be encouraged.
6. Connected Everything will strive to achieve diversity in event speakers and participants, for example:
   1. strive for gender balance and diverse ethnic representation for speakers for its events, conferences, webinars and podcasts.
   2. across all our activities, whether online or in person, written materials and outputs (podcasts, blogs, web pages) we will strive for diverse representation.
   3. whether Connected Everything activities are in person or online, we will strive to ensure they are accessible and we will aim to provide the right environment to allow everyone to participate.
7. Connected Everything leadership team will share its learning on EDI processes through:
   1. Articles/blogs/podcasts on its website
   2. Sharing with partners including other funded projects & Networks of Networks
   3. Writing a formal piece for publication in Q1 2021
   4. Evaluate its EDI processes and report this publicly, sharing its progress against the goals.

# **Table AI 1** Connected Everything selection criteria for stage 1 and 2 of the anonymous review process.

| **Stage** | **Criteria** |
| --- | --- |
| **Stage 1** | - Multidisciplinary and clear demonstration of “discipline-bridging” through activity - Involvement of industry stakeholders - Potential for development of future funding applications - Demonstration of transfer of concepts from other domains to manufacturing - Potential to lead to strong dissemination materials - Paths to accelerate impact of research to ensure rapid transfer to industry - Context, aim and objectives - Clear tangible deliverables - Appropriateness of the proposed methodology - Outline project plan |
| **Stage 2** | - Multidisciplinary and clear demonstration of “discipline-bridging” through activity - Involvement of ECR & industry stakeholders - Demonstration of transfer of concepts from other domains to manufacturing - Explore the novelty of the proposal - Potential to lead to strong dissemination materials - Paths to accelerate impact of research to ensure rapid transfer to industry - How have they considered responsible innovation in their proposed activity? - What are the keys risks in the project? - Explore the project plan in more detail - Potential for development of future funding applications |
